# Flow-Controlled Ventilation as a Rescue Strategy in Advanced COVID-19 ARDS: A Retrospective Observational Study

**DOI:** 10.3390/jcm15124439

**Published:** 2026-06-08

**Authors:** Meltem Ceylan Delice, Nilgun Kavrut Ozturk

**Affiliations:** 1 Department of Anesthesiology and Reanimation 1, SBU Van Training and Research Hospital, Van 65000, Türkiye; 2 Department of Anesthesiology and Reanimation 2, SBU Antalya Training and Research Hospital, Antalya 07100, Türkiye; nilgun.kavrutozturk@sbu.edu.tr

**Keywords:** ARDS, COVID-19, flow-controlled ventilation, gas exchange, lung-protective ventilation, mechanical ventilation, intensive care unit

## Abstract

**Background:** Acute respiratory distress syndrome (ARDS), particularly in COVID-19–related severe respiratory failure, remains a major challenge in intensive care. Flow-controlled ventilation (FCV) may improve gas exchange by enabling precise airway pressure control; however, clinical data on its prolonged use in ARDS are limited. **Methods:** This single-center retrospective observational study included adult patients with moderate to severe ARDS who underwent FCV during invasive mechanical ventilation. FCV was delivered using the Evone^®^ ventilator with the Tritube^®^. Demographic data, ventilatory settings, and arterial blood gas values were analyzed before and during 48 h of FCV and for 8 h after transition to conventional ventilation. **Results:** Seven patients with COVID-19–related ARDS were included. Following initiation of FCV, PaO_2_ increased within the first 8 h (median increase: +24 mmHg), accompanied by a median 38% improvement in the PaO_2_/FiO_2_ ratio, which remained above baseline throughout follow-up. Arterial PCO_2_ progressively declined, with the most pronounced reduction observed within the first 24 h (median decrease: −14 mmHg; approximately 22%). After transition back to conventional ventilation, mild deterioration in gas exchange parameters was observed; however, none returned to baseline values. All patients died during their ICU stay, mainly due to secondary infections and pulmonary embolism. **Conclusions**: In advanced COVID-19–related ARDS unresponsive to conventional ventilation, prolonged FCV application was technically feasible under controlled ICU conditions and associated with descriptively observed improvements in gas exchange parameters. However, late initiation of FCV did not translate into survival benefit. Prospective studies are required to define the optimal timing and patient selection for FCV. The present findings primarily support the technical feasibility and short-term physiological effects of FCV rather than clinical efficacy.

## 1. Introduction

ARDS remains a major challenge in intensive care practice, particularly in patients with COVID-19–related severe hypoxemic and mixed-type respiratory failure. Despite the use of lung-protective ventilation strategies, including low tidal volumes and optimized positive end-expiratory pressure (PEEP), adequate oxygenation and carbon dioxide elimination may remain difficult to achieve in a subset of patients.

FCV is a novel ventilation mode characterized by constant inspiratory and expiratory flow, enabling precise control of airway pressures, more homogeneous ventilation distribution, and potentially improved gas exchange while limiting ventilator-induced lung injury. Although experimental and early clinical data suggest beneficial physiological effects in ARDS, clinical experience with FCV in COVID-19 patients is still limited [1,2,3].

Although the exact mechanisms underlying the potential benefits of FCV in ARDS are still being investigated, several physiological properties may explain its favorable effects on gas exchange and lung protection. Unlike conventional ventilation modes characterized by passive expiration and variable expiratory flow patterns, FCV provides constant and actively controlled inspiratory and expiratory flow throughout the respiratory cycle. This approach may reduce dissipated mechanical energy, minimize cyclic alveolar opening and closing, and promote more homogeneous alveolar recruitment. By limiting abrupt pressure variations and improving expiratory flow control, FCV may reduce atelectrauma and mechanical power delivery to injured lungs. In addition, more efficient carbon dioxide elimination has been observed during FCV despite lower minute ventilation requirements, suggesting improved ventilatory efficiency and reduced dead-space ventilation. These physiological characteristics make FCV an attractive investigational strategy in severe ARDS, particularly in patients with refractory hypercapnia or heterogeneous lung involvement.

These characteristics may be particularly relevant in COVID-19–related ARDS, where heterogeneous regional lung involvement and prolonged ventilatory exposure frequently complicate conventional ventilation strategies. This retrospective observational study aimed to evaluate the physiological effects and technical feasibility of prolonged FCV application in patients with advanced COVID-19–related ARDS who remained unresponsive to optimized conventional lung-protective ventilation strategies.

## 2. Materials and Methods

### 2.1. Study Design and Setting

This study was designed as a retrospective, single-center observational study conducted in the intensive care unit of a tertiary-care university hospital. Adult patients treated between March 2021 and April 2022 with COVID-19 infection confirmed by polymerase chain reaction (PCR) and clinical findings and diagnosed with ARDS were included in the study and ventilated using an FCV mode with an Evone® ventilator (Ventinova Medical B.V., Eindhoven, The Netherlands).

ARDS was defined according to the Berlin criteria, and patients with moderate to severe ARDS under invasive mechanical ventilation were evaluated. Patients were consecutively included if they exhibited persistent hypoxemia and/or hypercapnia despite standard lung-protective ventilation strategies, including low tidal volume ventilation, appropriate PEEP, and adjunctive therapies such as prone positioning when indicated.

At the time of this clinical experience, extracorporeal membrane oxygenation (ECMO) was not routinely available in our institution for severe COVID-19 ARDS management. Therefore, FCV was considered a rescue ventilatory strategy in patients with refractory gas exchange abnormalities despite optimized conventional ventilation.

The decision to initiate FCV was made by the attending intensivist based on persistent refractory gas exchange abnormalities despite optimization of conventional ventilatory management. Lung ultrasound was used as a supportive bedside assessment tool, and findings consistent with diffuse aeration loss and consolidation were considered together with clinical and gas exchange parameters during the decision-making process for FCV initiation. To reduce selection bias, all consecutive patients who received FCV during the study period and fulfilled the inclusion criteria were included in the analysis. No patients were excluded after FCV initiation. A comparator group was not available due to the exploratory and feasibility-oriented nature of this clinical experience.

This study was designed to provide descriptive physiological and feasibility observations regarding prolonged FCV application in severe COVID-19–related ARDS. Accordingly, the findings should be interpreted as exploratory and hypothesis-generating rather than evidence of causal clinical benefit.

Patients were eligible for inclusion if they met all of the following criteria:

- Age ≥ 18 years
- Confirmed COVID-19 infection based on polymerase chain reaction (PCR) and compatible clinical findings
- Diagnosis of moderate to severe ARDS under invasive mechanical ventilation (ARDS severity was classified according to the Berlin definition as follows: Moderate ARDS: PaO_2_/FiO_2_ 100–200 mmHg with PEEP ≥ 5 cmH_2_O, Severe ARDS: PaO_2_/FiO_2_ < 100 mmHg with PEEP ≥ 5 cmH_2_O)
- Persistent hypoxemia and/or hypercapnia accompanied by respiratory acidosis despite optimized conventional lung-protective ventilation strategies, including low tidal volume ventilation, appropriate PEEP titration, and adjunctive therapies such as prone positioning when indicated
- Initiation of FCV as a rescue ventilatory strategy.

Patients were excluded if they were younger than 18 years, had no confirmed COVID-19 infection, had mild ARDS (PaO_2_/FiO_2_ ≥ 200 mmHg), were not receiving invasive mechanical ventilation, did not undergo FCV, or had incomplete clinical or ventilatory data.

### 2.2. Ventilation Strategy

Before FCV initiation, all patients were managed according to institutional COVID-19 ARDS protocols, including lung-protective ventilation, individualized PEEP titration, prone positioning, corticosteroid therapy, antimicrobial treatment when indicated, thromboprophylaxis, and nutritional support. Despite optimized conventional ventilation and prone positioning, persistent hypercapnia and refractory gas exchange abnormalities prompted consideration of FCV as a rescue strategy. FCV was delivered using the Evone^®^ ventilator (Ventinova Medical B.V., Eindhoven, The Netherlands) in combination with the Tritube^®^, a small-bore cuffed endotracheal tube specifically designed for FCV.

During FCV, a fixed inspiratory-to-expiratory (I) ratio of 1:1 was applied in all patients. Initial flow rates ranged between 16 and 20 L/min, and tidal volumes targeting approximately 6–8 mL/kg predicted body weight were adjusted according to respiratory mechanics and arterial blood gas analysis. PEEP levels were individualized using oxygenation response, dynamic respiratory compliance, driving pressure, and arterial blood gas measurements.

Ventilator monitoring included tidal volume, airway pressures, driving pressure, peak pressure, mean airway pressure, inspiratory and expiratory flow characteristics, and arterial blood gas parameters.

Arterial blood gas analyses were routinely obtained before FCV initiation, at the first hour following FCV initiation, and subsequently at regular intervals according to clinical status and ventilatory stability.

All patients received deep sedo-analgesia and continuous neuromuscular blockade with rocuronium infusion during FCV to ensure controlled ventilation and minimize patient–ventilator asynchrony.

Prone positioning had already been applied during the conventional ventilation period according to institutional ARDS management protocols, generally for at least 8–12 h daily when tolerated. During FCV, patients were mainly maintained in supine or alternating lateral positions to facilitate device management and monitoring stability.

Hemodynamic monitoring included continuous arterial blood pressure measurement, heart rate monitoring, vasopressor requirements, urine output, and serial arterial blood gas analysis. Clinically significant hemodynamic instability attributable to FCV was not observed during monitoring.

Special attention was given to secretion management because of the small internal diameter of the Tritube^®^. Active humidification, regular airway assessment, and careful suctioning procedures were routinely performed throughout FCV application.

FCV duration was limited to a maximum of 48 h. Transition back to conventional ventilation was performed following discontinuation of neuromuscular blockade or according to clinical judgment.

### 2.3. Data Collection

Demographic characteristics, clinical variables, ventilatory parameters, and arterial blood gas values were recorded at predefined time points: immediately before FCV initiation (baseline), and at 1 h, 24 h, and 48 h after the initiation of FCV. Typical FCV settings included constant inspiratory and expiratory flow, with adjustments guided by arterial blood gas analysis and airway pressure monitoring.

Parameters included oxygenation indices (PaO_2_, PaO_2_/FiO_2_ ratio), carbon dioxide levels (PaCO_2_), and arterial pH. Changes in oxygenation and carbon dioxide elimination over time were evaluated descriptively.

### 2.4. Ethical Considerations

Ethical approval for this study was obtained from the Antalya Training and Research Hospital Medical Research Scientific Ethics Committee (Antalya, Türkiye) (Approval No: 2026-6; Decision No: 1/8; 8 January 2026). The study was conducted in accordance with the Declaration of Helsinki. Due to the retrospective design of the study and the use of anonymized clinical data, the requirement for informed consent was waived by the ethics committee.

### 2.5. Statistical Analysis

Due to the small sample size and exploratory nature of this retrospective observational study, statistical analysis was limited to descriptive methods. Continuous variables are presented as median and interquartile range (IQR) or mean ± standard deviation where appropriate. No formal comparative or inferential statistical analysis was performed because of the absence of a control group and the limited sample size. Accordingly, the findings should be interpreted as descriptive physiological observations intended primarily for hypothesis generation. Given the rarity of prolonged FCV application in severe COVID-19 ARDS, the currently available clinical experience remains limited to small physiological and feasibility-oriented cohorts.

### 2.6. AI Statement

ChatGPT (GPT-5.5, OpenAI, San Francisco, CA, USA) was used exclusively for language editing, grammar correction, and improvement of manuscript readability. All AI-generated outputs were critically reviewed, revised, and approved by the authors, who take full responsibility for the final content.

## 3. Results

Seven patients were included in the analysis. The mean age was 68 ± 9 years. The median ICU length of stay before FCV initiation was 10 days (IQR 9–12), and the median total ICU length of stay was 19 days (IQR 16–22). At ICU admission, disease severity was high, with a median APACHE II score of 24 (IQR 22–28) and a median SOFA score of 10 (IQR 9–12), consistent with advanced critical illness and multi-organ dysfunction.

Following the initiation of FCV, PaO_2_ levels demonstrated a numerical increase within the first 8 h and remained above baseline throughout the observation period. The P/F ratio demonstrated an early favourable trend and remained relatively stable between 24 and 48 h, with no patients returning to baseline values during follow-up.

During FCV, a fixed inspiratory-to-expiratory (I:E) ratio of 1:1 was used in all patients. Initial flow rates ranged between 16 and 20 L/min, and tidal volumes were approximately between 450 and 530 mL.

**Figure 1:**
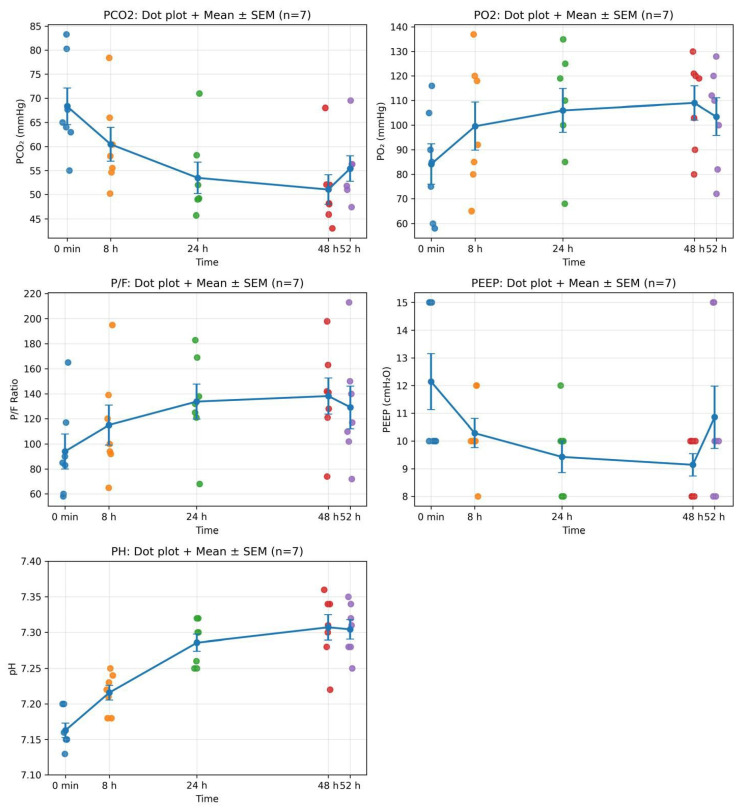
Time course of respiratory and blood gas parameters in ARDS patients treated with FCV.

**Figure 2:**
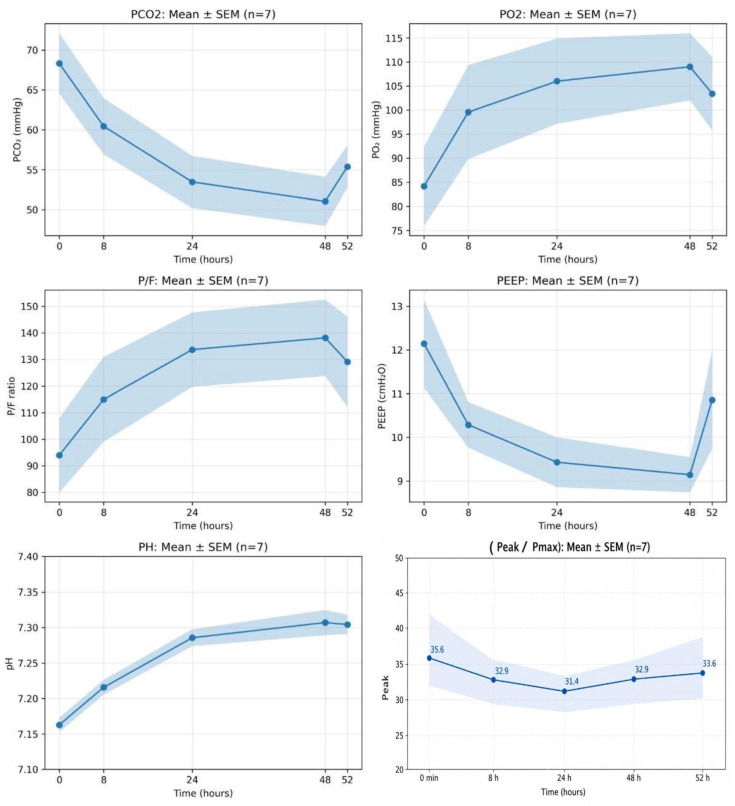
Temporal changes in PaO_2_/FiO_2_ ratio, PaCO_2_, arterial pH, and PEEP values during prolonged FCV application and after transition back to conventional ventilation in individual patients (P1–P7).

PCO_2_ levels demonstrated a gradual numerical decline, with the most pronounced reduction observed within the first 24 h. During the 24–48 h monitoring period, relative stabilization was noted. After the ventilatory mode change at 48 h, a limited increase in PCO_2_ was observed in some patients, accompanied by mild fluctuations in PaO_2_ and P/F ratio; however, none of the parameters returned to baseline levels. Ventilator settings and lung-protective parameters during FCV are presented in Figure 1 and Figure 2. Despite descriptively observed physiological improvements, no survival benefit was observed in this highly selected cohort with advanced refractory ARDS. The primary causes of death were secondary infections and pulmonary embolism.

Serial measurements of arterial carbon dioxide partial pressure (PCO_2_), PaO_2_/FiO_2_ ratio (P/F ratio), positive end-expiratory pressure (PEEP), and arterial pH are shown for each patient (P1–P7) at predefined time points during FCV and after transition to conventional ventilation. Symbols indicate sequential measurement time points. Individual patient trajectories demonstrated interindividual variability but consistent overall improvement in oxygenation and carbon dioxide elimination during FCV.

## 4. Discussion

One of the most notable aspects of our observations is the prolonged continuous application of FCV for up to 48 h, a duration longer than those reported in most previous human studies. Existing clinical investigations of FCV in ARDS have primarily focused on short-term physiological assessments, typically limited to minutes or up to 30 min [2,3]. In this context, extended FCV application appears technically feasible under controlled ICU conditions in selected patients. To our knowledge, this report represents one of the longest continuous applications of FCV described in critically ill COVID-19 ARDS patients.

In this evaluation, temporal changes in PaO_2_, PCO_2_, and the P/F ratio were analysed collectively across seven patients. Across the cohort, a marked increase in PaO_2_ and the P/F ratio was observed within the first 8 h, suggesting improved alveolar recruitment and ventilation–perfusion matching. During the same early period, PCO_2_ levels began to decline; however, this reduction was relatively modest, with full stabilization of ventilatory efficiency becoming evident within the first 24 h.

Between 8 and 24 h, improvements in oxygenation and the P/F ratio were sustained and accompanied by a more pronounced and consistent reduction in PCO_2_, suggesting that this interval represents the phase of maximal physiological benefit in terms of both oxygenation and ventilatory effectiveness.

During the 24–48 h follow-up period, PaO_2_, PCO_2_, and the P/F ratio demonstrated relative stabilization, reflecting maintenance of the early physiological gains. Following the change in ventilatory mode after 48 h, the subsequent 8 h assessment revealed a limited increase in PCO_2_ and minor fluctuations in PaO_2_ and the P/F ratio in some patients; however, none of these changes approached baseline values.

Overall, these findings suggest that the applied ventilatory strategy exerts a very early effect on oxygenation (within the first 8 h), followed by a slightly delayed but more sustained improvement in ventilatory efficiency (within the first 24 h). The observed interindividual variability underscores the heterogeneous nature of pulmonary involvement and highlights the importance of individualized ventilatory management in this patient population.

Stable patient–ventilator interaction during FCV was achieved under sedation and continuous neuromuscular blockade, consistent with previous reports indicating that FCV is best applied during fully controlled ventilation [3,4]. The treatment protocol recommended in ICU practice during the pandemic period appeared compatible with the operational requirements of FCV, allowing uninterrupted ventilation without clinically relevant asynchrony. During FCV, improvements in oxygenation and effective carbon dioxide elimination at comparatively lower airway pressures were observed. These findings are in line with experimental and clinical data demonstrating enhanced ventilation efficiency, reduced minute ventilation requirements, and improved lung recruitment under FCV [1,5].

Prolonged FCV application in the ICU also involves a relevant technical and organizational learning curve. Continuous staff familiarity with Tritube handling, humidification management, secretion control, and close respiratory monitoring is required to maintain ventilation safety and stability. In contrast to most previously published short-term operating room applications, prolonged ICU use of FCV necessitates sustained multidisciplinary monitoring and experienced personnel.

Flow-controlled ventilation was not applied as the initial ventilation strategy following intubation in the studied patient population. Instead, patients were managed with conventional mechanical ventilation for approximately five days before FCV initiation, most often in the setting of progressive moderate-to-severe ARDS, prolonged ventilatory exposure, and refractory gas exchange. Consequently, FCV was introduced at a relatively advanced stage of respiratory failure, which represents an important contextual factor when interpreting clinical outcomes.

Importantly, this study was not designed to evaluate survival benefit. The cohort represented a highly selected population with advanced refractory COVID-19 ARDS, prolonged exposure to conventional mechanical ventilation, severe parenchymal injury, and evolving systemic complications. Under these conditions, physiological improvement in gas exchange alone was unlikely to reverse the overall disease trajectory. Therefore, the present observations should be interpreted primarily as physiological and feasibility findings rather than evidence of clinical efficacy.

Under these conditions, the observed effects of FCV were largely confined to improvements in physiological parameters rather than meaningful modification of the overall ARDS trajectory or patient survival. This observation is consistent with the pathophysiology of COVID-19–related ARDS, which is characterized by diffuse and extensive lung injury, particularly in advanced disease stages, where prevention of ventilator-induced lung injury alone may be insufficient to reverse established structural damage [6]. Nevertheless, available experimental and clinical evidence suggests that earlier application of FCV—prior to the development of refractory carbon dioxide retention—may offer greater protective potential. In clinical practice, however, FCV is most often reserved for patients in whom adequate oxygenation and CO_2_ clearance cannot be achieved despite optimized conventional ventilation.

After 48 h of FCV, ventilation was transitioned to pressure-regulated volume control (PRVC), and patients were monitored for an additional 8 h observation period. This transition enabled assessment of whether the physiological benefits observed during FCV were strictly mode-dependent or partially sustained following return to conventional ventilation. While several ventilatory parameters tended to shift toward pre-FCV values, not all improvements were completely lost, suggesting a transient carry-over effect. Experimental ARDS models and ex vivo lung perfusion studies have demonstrated that FCV promotes more homogeneous lung aeration and recruitment, particularly in dependent lung regions, which may partly explain the partial persistence of gas exchange improvements after mode transition [1,7].

A pneumothorax occurred in one patient (Patient 7) during FCV at 32 h despite lower peak airway pressures compared with PRVC. Pneumothorax is a recognized complication in advanced ARDS and COVID-19–related lung injury. Given the severity of underlying parenchymal damage and existing evidence that FCV reduces mechanical power and dissipated energy, this event is most plausibly attributable to disease-related lung fragility rather than a device-associated effect [5,8].

All patients were managed according to a standardized ICU protocol that included prone positioning for at least 8 h per day from the first day of ICU admission. During FCV assessment, positioning was limited to right and left lateral positions to ensure device compatibility and measurement stability. Carbon dioxide values obtained via tracheal measurements were cross-validated with arterial blood gas analysis. Despite theoretical concerns regarding increased mean airway pressure during FCV, no clinically significant hemodynamic instability (>10% variation) was observed, consistent with prior human studies reporting acceptable hemodynamic tolerance during FCV [3].

Finally, the device’s integrated monitoring capabilities, including continuous visualization of multiple respiratory parameters and CO_2_-related metrics, represent a technological advantage that may facilitate individualized ventilatory adjustments in complex ARDS cases. Future investigations should evaluate earlier FCV initiation, comparative efficacy against conventional pressure-controlled ventilation modes, mechanical power reduction during prolonged ICU ventilation, imaging-based assessment of regional lung recruitment, and identification of ARDS phenotypes most likely to benefit from FCV.

Larger prospective and randomized clinical trials are required to define optimal timing, duration, and patient selection for FCV and to clarify its role within lung-protective ventilation strategies.

This study has several important limitations. First, the retrospective single-center design and small sample size (n = 7) limit the generalizability of the findings. Second, the absence of a control group prevents any causal inference regarding the effects of FCV. Third, FCV was introduced after a period of conventional mechanical ventilation in patients with moderate to severe ARDS, which may have influenced clinical outcomes. In addition, the potential effects of concomitant therapies and disease progression cannot be excluded, and the observed physiological improvements cannot be attributed solely to FCV. Finally, the small internal diameter of the Tritube may also increase airway resistance and complicate secretion clearance during prolonged ventilation. In addition, it poses a theoretical risk of airway obstruction from secretions, and the use of FCV limits the application of certain adjunctive therapies, such as inhaled anesthetics or nitric oxide, potentially reducing flexibility in clinical practice.

## 5. Conclusions

In this retrospective observational study of patients with advanced COVID-19–related ARDS unresponsive to conventional ventilation strategies, prolonged application of FCV was technically feasible and consistently associated with early and sustained improvements in oxygenation and carbon dioxide elimination. These physiological benefits were most pronounced within the first 24 h and were partially maintained even after transition back to conventional ventilation.

Despite these favourable respiratory effects, the absence of survival benefit in this cohort highlights the critical importance of timing. FCV was introduced at a late stage of disease, when extensive lung injury and systemic complications were already established, likely limiting its impact on overall clinical outcomes. Therefore, while FCV appears to be a potential adjunctive strategy for improving gas exchange, its role may be more meaningful if applied earlier in the course of ARDS.

Future prospective, randomized studies are essential to determine the optimal timing, duration, and patient selection for FCV and to clarify whether its physiological advantages can translate into improved clinical outcomes. Accordingly, these findings should be considered hypothesis-generating and primarily supportive of physiological feasibility rather than definitive clinical benefit.

## Data Availability

The datasets used and/or analysed during the current study are available from the corresponding author on reasonable request.

## Abbreviations

The following abbreviations are used in this manuscript:

ARDS	Acute respiratory distress syndrome
FCV	Flow-controlled ventilation
PEEP	Positive end-expiratory pressure

## References

[1] Enk D., Spraider P., Abram J., Barnes T. (2020). Pressure measurements in flow-controlled ventilation. Crit. Care Med..

[2] Weber J., Straka L., Borgmann S., Schmidt J., Wirth S., Schumann S. (2020). Flow-controlled ventilation (FCV) improves regional ventilation in obese patients: A randomized controlled crossover trial. BMC Anesthesiol..

[3] Van Dessel E.D., De Meyer G.R., Morrison S.G., Jorens P.G., Schepens T. (2022). Flow-controlled ventilation in moderate acute respiratory distress syndrome due to COVID-19: An open-label repeated-measures controlled trial. Intensive Care Med. Exp..

[4] Spraider P., Putzer G., Breitkopf R., Abram J., Mathis S., Glodny B., Martini J. (2021). A case report of individualized ventilation in a COVID-19 patient: New possibilities and caveats to consider with flow-controlled ventilation. BMC Anesthesiol..

[5] Barnes T., van Asseldonk D., Enk D. (2018). Minimisation of dissipated energy in the airways during mechanical ventilation by using constant inspiratory and expiratory flows: Flow-controlled ventilation (FCV). Med. Hypotheses.

[6] Gattinoni L., Tonetti T., Quintel M. (2017). Regional physiology of ARDS. Crit. Care.

[7] Ordies S., Orlitova M., Heigl T., Sacreas A., Van Herck A., Kaes J., Neyrinck A.P. (2020). Flow-controlled ventilation during EVLP improves oxygenation and preserves alveolar recruitment. Intensive Care Med. Exp..

[8] Van Oosten J.P., Francovich J.E., Somhorst P., van der Zee P., Endeman H., Gommers D.A.M.P.J., Jonkman A.H. (2024). Flow-controlled ventilation decreases mechanical power in postoperative ICU patients. Intensive Care Med. Exp..

